# All-in-one generation and multiomic profiling of human whole brain organoid on a millifluidic plate

**DOI:** 10.1016/j.mtbio.2025.102653

**Published:** 2025-12-09

**Authors:** Wen Zhao, Yu Wang, Tao Chen, Min Shen, Jibo Wang, Xuemei Huang, Lili Zhu, Ting Yu, Zhentao Zhang, Yunhuang Yang, Maili Liu, Dong Wang, Weihua Huang, Rui Hu, Pu Chen

**Affiliations:** a State Key Laboratory of Metabolism and Regulation in Complex Organisms, Taikang Medical School (School of Basic Medical Sciences), Taikang Center for Life and Medical Sciences, Wuhan University, Wuhan, 430071, China; b Tissue Engineering and Organ Manufacturing (TEOM) Lab, Department of Biomedical Engineering, Wuhan University TaiKang Medical School (School of Basic Medical Sciences), Wuhan, 430071, China; c State Key Laboratory of Magnetic Resonance and Atomic and Molecular Physics, National Center for Magnetic Resonance in Wuhan, Innovation Academy for Precision Measurement Science and Technology, Chinese Academy of Sciences-Wuhan National Laboratory for Optoelectronics, Huazhong University of Science and Technology, Wuhan, 430071, China; d University of Chinese Academy of Sciences, Beijing, 100049, China; e State Key Laboratory of Digital Medical Engineering, Key Laboratory of Biomedical Engineering of Hainan Province, Biomedical Engineering School of Hainan University, Haikou, 570228, China; f Department of Neurology, Renmin Hospital of Wuhan University, Wuhan, 430050, China; g College of Chemistry and Molecular Sciences, Wuhan University, Wuhan, 430072, China; h Brain Research Center, Zhongnan Hospital of Wuhan University, Wuhan, 430071, China

**Keywords:** Brain organoid, Organ-on-a-chip, Multiomic profiling, Millifluidic plate, Long-term culture

## Abstract

Human brain organoids (hBOs) have been recently regarded as neurobiologically relevant brain models and exponentially exploited in a variety of neuroscience research. However, the current gold-standard method for generating hBOs is intricate and laborious, resulting in hBOs with morphological variability and inconsistent batch-to-batch reproducibility. Despite several studies reporting simplified hBO culture methods, few of those methods was biologically validated with multiomic profiling, which is crucial for neurobiological studies. Here, we demonstrate an all-in-one millifluidic plate (AIOMP) with individually perfusable microchambers for hBOs, simplifying the culture process, improving the uniformity and reproducibility, and enabling long-term cultivation and real-time morphogenesis observation. Additionally, our comprehensive transcriptomic and proteomic analyses revealed that AIOMP increases neurogenesis and corticogenesis of hBOs, suggesting a stronger correlation between the AIOMP-generated hBOs and human fetal brain than those generated through conventional method. Metabolomic and neurophysiological results further support the maturation-enhancing effects of AIOMP on hBOs, showing improved neurotransmitter synthesis and electrophysiological functionality. Overall, the AIOMP approach offers a simplified, reproducible, and biologically validated method for hBOs generation and maturation, with potential applications in neurobiology, neurological disease research, and central nervous system drug assessment.

## Highlights

• An AIOMP was developed to simplifying the culture procedure and improving the morphological homogeneity of hBOs.
• The microenvironment within AIOMP promoted neurotransmitter synthesis and electrophysiological function in hBOs.
• Multiomics analysis confirmed that AIOMP can drive the early-stage corticogenesis and neurogenesis of hBOs.

## Graphical abstract

**Figure:**
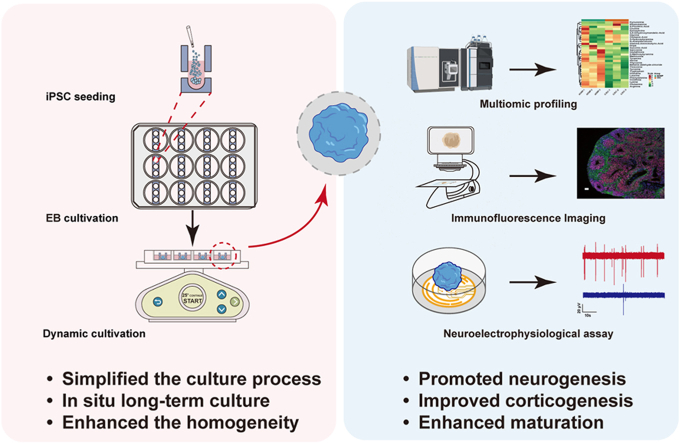

## 1 Introduction

Human brain organoids (hBOs) have been increasingly recognized by neurobiologists and neurologists as a transformative brain research model system in recent years [[1], [2], [3], [4]]. hBOs initially differentiated from human pluripotent stem cells (hPSCs) emulate parts of human neurodevelopmental trajectory and ultimately resemble some critical features of human brain relevant characteristics, including brain regionalization, brain region-specific cytoarchitecture and neural types, and neuro-electrophysiological activities [[5], [6], [7]]. Given the distinct differences between human brains and conventional brain research models, namely two-dimensional (2D)/three-dimensional (3D) neuronal culture models and transgenic model animals [[8], [9], [10]], hBOs hold a better promise to address long-lasting challenges in unveiling molecular basis of human neurological disease and identifying effective drug candidate for neurological disorders under the context of human neurobiologically and neurologically relevant models [5,11,12]. Therefore, hBOs have been rapidly employed in a broad range of studies not limited to neurobiology, neurological diseases, and CNS drug assessment [[13], [14], [15]]. Particularly, hBOs notably facilitate viral research [16] and have been used to identify mechanisms of viral entry, replication, and tropism during Zika and SARS-CoV-2 epidemics [[17], [18], [19], [20], [21]]. Additionally, hBOs have also been used to probe mechanisms of neurological disorders [[22], [23], [24]], including microcephaly [3], schizophrenia [25], traumatic brain injury [26] and Alzheimer's disease [[27], [28], [29]], and provided important insights into the onset and progression of pathogenesis.

Currently, the conventional standard culture (CSC) approach to generating hBOs follows the principle of neurodevelopmental biology. The generation of hBOs starts from hPSCs and experiences complex parenchymal cell fate transitions from ectoderm, neuroectoderm, neuroepithelium, neural stem cells, and unmatured neurons to matured neurons. The hBOs differentiation procedure demands laborious operation steps on multiple devices, generally including the generation of embryonic bodies on a 96-well U-shaped low adhesion plate, neuroectoderm induction on a 24-well plate, Matrigel embedding, neuroepithelial bud expansion in a 6-well plate, and suspension culture in a 6-well plate or a bioreactor [30]. Notably, the suspension culture is conducted in a bulk flow with a high Reynold number [31,32]. It lacks precise microenvironment control over individual organoids, which results in increased intra-batch morphological heterogeneity and lower inter-batch reproducibility [33]. This issue poses a great challenge for quantitative neural studies such as neuroprotectant screening and neurotoxicity assessment. Additionally, the suspension culture is inaccessible for in-situ and time-lapse observation of the same single organoids, which is essential to understand neurodevelopment and morphogenesis. Therefore, there is a strong demand for innovative brain organoid culture methods that allow simple operation steps and reproducible organoid formation.

Recent advances in converging development biology and microscale bioengineering permit simplifying hBOs formation and producing uniform hBOs under a tightly controlled microenvironment [[34], [35], [36], [37]]. Specifically, the cultivation of hBOs on a microfluidic chip, also called brain-organoid-on-a-chip, enables well-defined microenvironment controls over various biophysical and biochemical factors such as velocity, nutrient/waste exchange, and oxygen diffusion, which improves morphological uniformity and batch-to-batch reproducibility and even enhances neuronal specification and maturation [38]. Additionally, the millifluidic chip is compatible with optical microscopic observation and empowers in situ studies of neurodevelopment. However, millifluidic devices with close culture chambers confined hBOs growth in a restricted space and hampered hBOs growth and morphogenesis [39,40]. To solve this issue, a few recent studies introduced open-chamber millifluidic devices for hBOs culture. However, few of these millifluidic devices allows accommodate all culture steps on a single device [32,41]. More importantly, hBOs differentiation is highly sensitive to the local microenvironments, and bioengineered culture devices with varied geometric and hemodynamic designs may lead to distinct protein expression pattern and neurodevelopmental fate even under the same biochemical context [31]. Thus, it's essential to perform a fully biological validation of resulting hBOs before subsequent applications. However, all these studies only conduct simple biological characterization limited to cell viability, organoid morphology, and specific protein expression [[42], [43], [44]]. None of these studies provide full biological validation and characterization of hBOs fate at the transcriptome and proteome levels, which is critical for hBOs to become a standard model for widespread applications in neurobiology and neurological studies.

Here, we develop an all-in-one millifluidic plate (AIOMP) to generate hBOs with a facile procedure on a single device without transferring. The AIOMP is built in a 12-well plate, with each well containing three hBOs in three separate microchambers. The AIOMP allows reproducible formation of uniform hBOs and in-situ long-term observation of hBOs morphogenesis. We perform full biological characterization of hBOs using comprehensive transcriptome and proteome analysis in addition to RT-qPCR and immunofluorescence analysis. We find that the AIOMP exhibits a higher fidelity to the human fetal brain than CSC and promotes neurogenesis and corticogenesis to a great extent. Ultimately, we conducted neurotransmitter metabolism profiling and electrophysiological signal detection on long-term cultured hBOs. Our study demonstrated that AIOMP promotes the maturation of neurotransmitter synthesis and electrophysiological function in hBOs.

## 2 Experimental section/methods

### 2.1 Design of AIOMP

This design principle aims to generate human brain organoids (hBOs) through a straightforward procedure on a single device, allowing for the consistent creation of uniform hBOs and enabling the real-time observation of hBOs morphogenesis. The organoid chamber is specifically engineered to establish an isolated and regulated microenvironment for each brain organoid by being physically separated from other chambers and connected to the medium reservoirs. The U-shaped low-adhesion substrate within the chamber is intended to promote the clustering of human embryonic stem cells (hESCs) into embryonic bodies. Additionally, the tunnels that link to the side reservoirs facilitate bidirectional perfusion culture, which is crucial for sustaining a stable nutrient supply and efficient waste removal.

Dimension Selection: The dimensions of the organoid chamber, with a diameter of 5 mm and a height of 8 mm. These dimensions provide enough space for the growth and development of the brain organoids while ensuring that the organoids are not too large to cause flow disturbances or nutrient diffusion problems within the millifluidic system. A smaller diameter might limit the growth potential of the organoids, while a larger diameter could lead to difficulties in maintaining a uniform microenvironment. The height of 8 mm takes into account the need for sufficient medium exchange and the prevention of organoids from adhering to the top or bottom of the chamber.

### 2.2 Fabrication of AIOMP

The functional inserts were fabricated by using a water-cooling CO_2_ laser engraving system (350-50 W, Longtai laser, Liaocheng, China). Each insert had three circular chambers (4 mm diameter and 8 mm height) for culturing hBOs. Each chamber had two groups of 3 lateral holes (1 mm diameter) for medium perfusion. The inserts were thoroughly rinsed and soaked in ddH_2_O for 48 h. After drying, the inserts were integrated into microwells of 12-well plates. The PDMS prepolymer (RTV 615, Momentive, NY, USA) was mixed with a curing agent at a ratio of 10:1 (w/w) and utilized as an adhesive for sealing the gaps between inserts and microwells, followed by curing in an oven at 80 °C for 2 h. In addition, a small amount of PDMS was added to the chambers and cured to generate hydrophobic U-shape substrates. The assembled millifluidic plates were placed under ultraviolet light overnight for sterilization before cell seeding.

### 2.3 Numerical simulations

A numerical model based on the finite element method was established to predict the flow field in the AIOMP on rocking shaker and petri dish on orbital shaker. The numerical simulation was conducted on COMSOL Multiphysics (COMSOL Inc., Burlington, MA, USA). The “Laminar Flow” module was used to model the motion characteristics of the fluid in the plate, and the periodic motion of the AIOMP was modeled by using the “Dynamic Mesh, Rotating Domain”. Moreover, the rotating angle was set to 25°, and the rotation axis located at the lower midpoint of the chamber. The boundary conditions on air-liquid interface were set to “Open Boundary”. The problem was solved via a “Time-Dependent” solver.

Transient simulations were conducted for six distinct rocking periods (T = 0.5, 5, 10, 20, 50, and 100 s). Velocity data were extracted along a linear path a–a′ aligned with the central axis of the chamber, spanning from point a (bottom center) to point a′ (top center), covering the height to capture the most representative flow profile affecting organoid development (Fig. S2B). At the moment of maximum tilt angle (25°) in each cycle, velocity magnitudes were sampled at 100 equidistant points along this path using COMSOL's Line Graph feature. The raw velocity datasets for each rocking condition were exported to OriginLab (Origin 2022) for subsequent processing. The raw velocity data underwent normalization and logarithmic transformation (base-10 logarithm) to enhance comparability across different rocking periods. This processing enabled a clear comparison of flow dynamics under varying culture conditions.

### 2.4 Human PSC maintenance

Human H9 ESC lines were maintained in Nuwacell ncTarget hPSC Medium (RP01020, Nuwacell Biotechnologies), passaged via StemPro® Accutase (A1110501, Gibco) detachment, and reseeded in a vitronectin (A31804, Gibco)-coated 6-well plate filled with hPSC Medium containing 10 μM Rho-associated protein kinase (ROCK) inhibitor Y-27632 (72304, STEMCELL).

### 2.5 Generation of hBOs

The hBOs in the CSC group were generated and cultured with a slightly modified protocol from a previously described method by Lancaster et al. [30]. Human H9 ESC clumps were dissociated by Accutase (A1110501, Gibco) to generate single cells. EBs were generated by seeding 3000 cells into a well of ultra-low-attachment 96-well plate (CLS7007, Corning). EBs were cultured in hESC-maintaining medium supplemented with 4 ng/mL bFGF (4114 TC-01M, R&D.) and 10 μM ROCK inhibitor (72304, STEMCELL). After 5–6 days of culture, the formed EBs were transferred into a low-attachment 24-well plate. To induce the neuroepithelium-like structures, the EBs were cultured in suspension in a neural induction medium composed of DMEM/F12, 1 × N2 supplement (17502048; Thermo Fisher Scientific), 1 × GlutaMAX (35050061, Thermo Fisher Scientific), 1 × MEM-NEAA (11140050; Thermo Fisher Scientific), and 1 mg/mL heparin (H3149-10KU, Sigma). The medium was exchanged daily for 5 days. On day 11, EBs with neuroepithelium identity were encapsulated in 4 μL Matrigel each organoid (354277, Corning) and then transferred to ultra-low-attachment 6-well plate (CT-3526, Corning) and were further cultured in a induction medium comprising DMEM/F12 (11320082, Thermo Fisher Scientific) and Neurobasal (21103049, Thermo Fisher Scientific) (1:1) ratio supplemented with 1:200 (v/v) N2 supplement (17502048, Thermo Fisher Scientific), 1:100 (v/v) B27 supplement without retinoic acid (12587010; Thermo Fisher Scientific), 1:100 (v/v) GlutaMAX (35050061, Thermo Fisher Scientific), 1:200 (v/v) MEM-NEAA (11140050, Thermo Fisher Scientific), 12.5 μL insulin (I9278, Sigma), and 3.5 μL/mL β-mercaptoethanol (21985023, Sigma-Aldrich) in the stationary condition for 4 days. On day 15, the plates were transferred to an orbital shaker (HS-25A, MIULAB) rotating continuously at 80 rpm. The culture medium in the dishes was replaced with the neural maturation medium. The neural maturation medium for the dynamic culture had the same composition, except for the B27 supplement with vitamin A (17504044, Thermo Fisher Scientific).

To generate hBOs on the AIOMP, the steps of differentiation are consistent with the traditional culture method as above. Initially, 3000 dissociated hESCs were seeded into each cultivation chambers within a 12-well plate. Suspended hESCs will spontaneously aggregate into EBs. After the EBs formed, we then embedded the EBs with Matrigel (4 μL each organoid), and subjected them to neural induction, followed by placing the AIOMP onto a bidirectional rocking platform set at a 25° rocking angle and cycle of 20s for continuous rocking inside an incubator. Medium change was performed every other day after Matrigel embedding. The consumed medium was completely aspirated and replaced with fresh medium, avoiding the need of direct pipetting on top of organoids during the traditional culture medium change process and related risks of loss, damage, and contamination of organoids by accidently pipetting organoids into the aspiration pipet. The hBOs were maintained in dynamic culture for 35 days and 100 days before we characterized them.

### 2.6 Morphological analysis

**Fig. 1:**
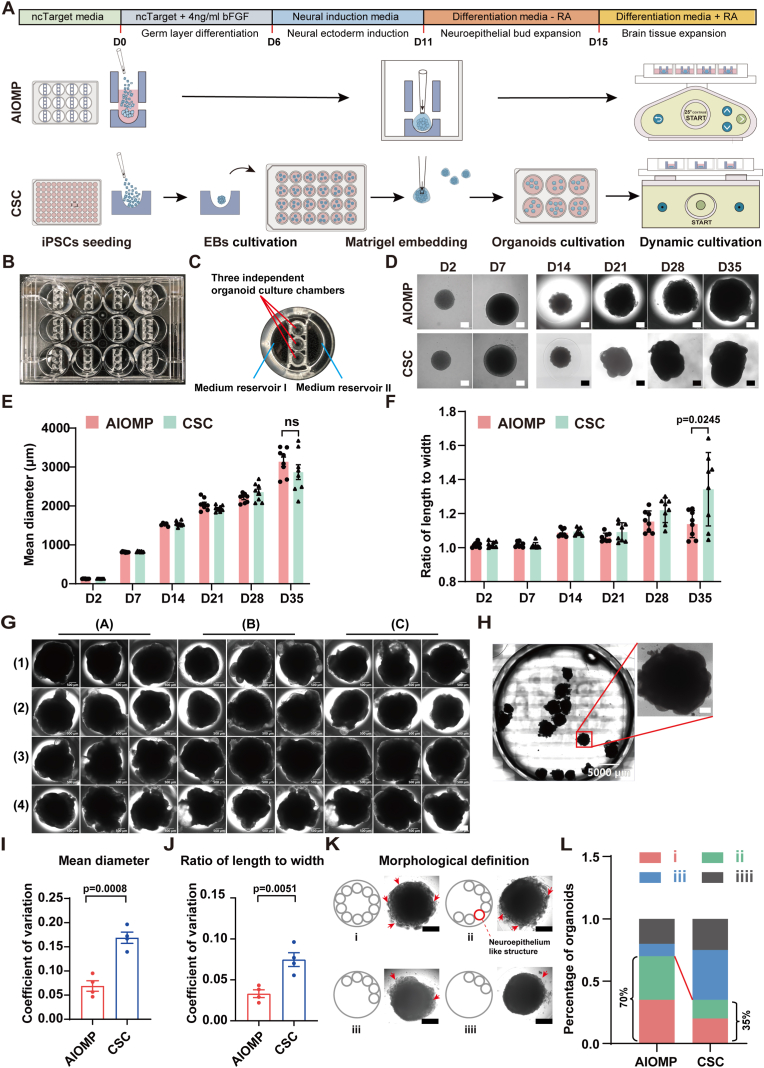
Evaluation of the homogeneity and reproducibility of brain organoid formation in the AIOMP. (A) Schematic diagram of conditions used to culture brain organoid by orbital shaker (traditional method) and by AIOMP. (B) The physical map of AIOMP. (C) Partial view of AIOMP single culture unit. (D) Brightfield images of brain organoids at different days. (Scale bar = 100 μm for D2 and D7; 500 μm for D14, D21, D28, and D35). (E, F) The diameter and the ratio of length to width of the brain organoids (n = 12). (G, H) Representative images of hBOs cultured in the AIOMP and CSC group at D35, respectively. (Scale bar = 500 μm for G; 5000 μm for H). (I, J) The variation of the diameter and the ratio of length to width of the hBOs in each condition at D35 (n = 4). (K) Schematic diagrams of the morphological assessment criteria based on the distribution of the neuroepithelium like structures: Type i, neuroepithelium like structure all around the aggregate; Type ii, neuroepithelium like structure more than three quarters around; Type iii, neuroepithelium like structure more than halfway around; Type iiii, neuroepithelial structure below the quarter around. (L) Percentages of organoids with each morphological type. AIOMP, n = 33; CSC, n = 30).

The hBOs at various culture stages in AIOMP and CSC (D2, D7, D14, D21, D28, D35, and D100) were imaged in two dimensions under bright-field conditions. ImageJ software was then employed to analyze average diameter, major and minor axes, and conduct morphological assessments. The ratio of the major to minor axes is defined as the ratio between the longest and shortest axes of the hBOs' projected area. The morphological analysis was performed by meticulously identifying structures with characteristics of neural tissue within D14 hBOs. Classification criteria were established based on the degree of development and the extent of coverage of neural tissue-related structures, categorizing hBOs into the following four types (Fig. 1K): Type i: Neural tissue-like structures are highly developed and completely cover the organoid's area. Type ii: Neural tissue-like structures cover more than three-quarters of the organoid's area, with some regions being slightly less developed, yet the main structures are relatively mature. Type iii: Neural tissue-like structures cover more than half but less than three-quarters of the organoid, with a certain range of immature areas present. Type iiii: Neural tissue-like structures cover less than a quarter of the organoid, with the majority of the area in undifferentiated region.

### 2.7 The thickness of CP layer

The CP thickness was analyzed systematically, following a privious reported method [3]. Frozen sections from the mid-region of each organoid were exclusively chosen for analysis to ensure data representativeness. Immunostaining for SOX2 and Tuj1 was performed on day 35 for both AIOMP and CSC groups, with subsequent imaging using a fluorescence microscope. The VZ was identified by specific SOX2 immunoreactivity and neural tube-like morphology. The CP layer was defined as the region between the VZ boundary and the outer surface showing Tuj1 expression. ImageJ software was used for precise thickness measurements. Three measurements were taken for each cortical structure at 45-degree angles to account for potential structural variability, with the average of these measurements calculated.

### 2.8 Histology and immunofluorescence

The 35-day old hBOs were washed gently three times with 1 × PBS and fixed with 4 % (w/v) PFA at 4 °C overnight. Fixed organoids were then transferred to 30 % (w/v) sucrose at 4 °C overnight. Then, Cryoprotected hBOs were then embedded with O.C.T compound (Sakura Finetek) at −40 °C. Embedded hBOs were sectioned into 10 μm thick slices with a cryostat (CM1950, Leica). The sections were then washed thoroughly three times with 1 × PBS. Blocking and permeabilization were performed using 0.1 % (w/v) saponin and 1 % (v/v) BSA in PBS for 1 h. The sections were incubated with primary antibodies at 4 °C overnight. Subsequently, the sections were incubated with the secondary antibodies according to Table S3. Images were captured on fluorescence microscope (IX-83, Olympus).

### 2.9 RNA extraction and reverse transcription quantitative PCR (RT-qPCR)

To detect the gene expression level of brain-specific genes, at least 3 hBOs (D35) were combined for a single sample of RNA extraction (n = 3, biological replicates = 3). Briefly, total mRNAs were isolated from the hBOs or the cells using Trizol reagent (B511311, Sheng Gong), and then cDNA was synthesized using ABScript Ⅲ RT Master Mix (RM21452, ABclonal Technology). RT-qPCR was performed using SYBR Green Real-time PCR Master Mix (RK21203, ABclonal Technology) under the following reaction conditions (35 cycles): denaturation at 95 °C for 1 min, annealing at 58 °C for 30 s, and extension at 72 °C for 30 s. Primer sequences were described in Table S2. The expression levels were normalized relative to the expression of the housekeeping gene GAPDH using the comparative Ct–method 2^−ΔΔCt^.

### 2.10 Protein extraction and trypsin digestion

The hBOs generated by AIOMP (n = 3, biological replicates = 3) and CSC groups (n = 3, biological replicates = 3) were collected at D35 for proteomic analysis. All organoids were washed 3 times with phosphate-buffered saline (PBS), then resuspended in PBS containing 1 mM phenylmethanesulfonylfluoride (PMSF), and crushed by sonication (3s on and 3s off) for 20 min with an output of 130 W on ice. The homogenized lysate was discarded by centrifugation at 6000 rpm for 20 min at 4 °C to discard cell debris, and the protein concentration of the resulting supernatant was measured using the bicinchoninic acid (BCA) method (Jiangsu Beyantian Institute of Biotechnology, China). Approximately 500 μg of protein was precipitated with 10 % trifluoroacetic acid (TFA) and 1 % sodium deoxycholate, then desalted three times with ice-cold acetone. After washing, the dried protein pellet was redissolved in 50 mM NH_3_HCO_3_. Then, all samples were reduced by adding 25 mM DL-dithiothreitol (DDT) at 37 °C for 45 min and alkylated with 50 mM iodoacetamide (IAA) at 25 °C for 30 min in the dark. For in-solution trypsin digestion, Trypsin was then added at 1:50 (w/w) at 37 °C for 12 h, and further complete digestion with trypsin (1:100 w/w) at 37 °C for 8 h, Finally, 0.1 % trifluoroacetic acid (TFA) was added to terminate the digestion and the solutions were desalted by a Strata X C18 SPE column (Phenomenex, Torrance, CA, USA) and vacuum-dried.

### 2.11 LC-MS/MS analysis

LC-MS/MS data acquisition was carried out on a Q Exactive HF-X mass spectrometer coupled with an Easy-nLC 1200 system (Thermo Fisher Scientific). The purified peptides were redissolved in 0.1 % formic acid (FA) and loaded onto a C18 trap column and then eluted into a C18 analytical column (75 μm × 25 cm, 2 μm particle size, 100 Å pore size, Acclaim PepMap C18 column, Thermo). Mobile phase A (0.1 % formic acid) and mobile phase B (80 % ACN, 0.1 % formic acid) were used to establish 90 min analysis gradient. A constant flow rate was set at 300 nL/min. For Data-Dependent Acquisition (DDA) mode analysis, each scan cycle consisted of one full-scan mass spectrum (R = 60 K, AGC = 3e6, max IT = 20 ms, scan range = 350–1800 *m*/*z*) followed by 25 MS/MS events (R = 15 K, AGC = 2e5, max IT = 50 ms). HCD collision energy was set to 28. Isolation window for precursor selection was set to 1.6 Da. Former target ion exclusion was set for 35s.

### 2.12 Database search and protein quantification

For the identification, all MS raw data were analyzed with MaxQuant (V1.6.6) using the Andromeda database search algorithm. Spectra files were searched against the UniProtKB human proteome database using the following parameters: LFQ mode was checked for quantification; Variable modifications, Oxidation (Met); Fixed modification, Carbamidomethylation (Cys); Enzyme specificity was set as full cleavage by trypsin with two maximum missed cleavage sites permitted. Search results were filtered with 1 % false discovery rate (FDR) at both protein and peptide levels. The mass tolerance was set ±20 ppm during the first search and ±4.5 ppm during the main search, proteins identified based on the presence of at least two unique peptides were used for reporting.

The protein quantification was achieved by using the LFQ algorithm in MaxQuant, and LFQ intensities were used for protein ratios calculation. Statistical evaluation of data was performed using Perseus software (version 1.6.15.0), and proteins identified and quantified in three biological replicates were used for relative quantification. For quantification, the raw intensities were normalized by the median value, and the average value of the 3 repeated ratios was taken as the relative quantification (ratio) of the final difference correction in the control group. A two-sample *t*-test to calculate the p value for statistical evaluation. The differentially expressed proteins (DEPs) were defined as fold change ≥1.5 or ≤ 0.67, p < 0.05, and coefficient of variation (CV) < 20 %.

### 2.13 Bioinformatics analysis

To further evaluate the biological function of DEPs, Gene Ontology (GO) annotation of Blast2GO software was used to classify the identified proteins into biological process (BP), cellular component (CC) and molecular function (MF). Online tools DAVID Bioinformatics Resources (https://david-d.ncifcrf.gov/) and Kyoto Encyclopedia of Genes and Genomes (KEGG, http://www genome.jp/kegg/) database were used for GO term function enrichment and protein pathway annotation. For statistical significance, the hypergeometric test and expressed as a p value, p < 0.05 was considered to be statistically significant.

### 2.14 RNA-seq transcriptomic analysis of RNA extraction

For sample preparation, hBOs from each condition (AIOMP/CSC, n = 3, biological replicates = 3) were collected on the D35. Total RNA was first isolated by TRIzol reagent (Invitrogen) according the manufacturer's instructions (Invitrogen) and genomic DNA was removed using DNase I (Takara). RNA concentration was measured using a ND-2000 (NanoDrop Technologies), and the integrity of total RNA was checked by 2100 Bioanalyser (Agilent). RNA-seq transcriptome libraries was prepared using 1 μg of total RNA following the TruSeqTM RNA sample Prep Kit from Illumina (San Diego, CA). In brief, mRNA was enriched according to polyA selection method by oligo(dT) magnetic beads with two rounds of the purification process and then fragmented by fragmentation buffer. The first strand of cDNA was synthesized from fragments of cleaved RNA using a SuperScript double-stranded cDNA synthesis kit (Invitrogen, CA) in the presence of random hexamer primers (Illumina). Afterward, a single ‘A’ nucleotide was then ligated to the 3′ end of the cDNA fragments followed by ligation of adapters according to Illumina's library construction protocol. To create the final strand-specific cDNA library, Libraries were size-selected against a 300 bp cDNA target fragment on 2 % Low Range Ultra Agarose, followed by 15 cycles of PCR amplification using Phusion DNA Polymerase (NEB). After quantified by TBS380, paired-end RNA-seq sequencing libraries were sequenced with the Illumina HiSeq xten/NovaSeq 6000 sequencer (2 × 150 bp read length).

### 2.15 Differential expression analysis and functional enrichment

To identify DEGs (differential expression genes) between hBOs samples cultured under different conditions (AIOMP/CSC), The expression levels for each transcript were calculated according to the transcripts per million (TPM) method and gene abundances were quantified using RSEM (http://deweylab.biostat.wisc.edu/rsem/). Essentially, differential expression analysis was performed using the DESeq2/DEGSeq/EdgeR with Q value ≤ 0.05, DEGs with |log2 Fold Change|>1 and Q value ≤ 0.05 (DESeq2 or EdgeR)/Q value ≤ 0.001 (DEGSeq) were considered to be DEGs. In addition, GO and KEGG functional enrichment analyzes were performed to identify which DEGs were significantly enriched in GO terms and metabolic pathways at Bonferroni-corrected P value ≤ 0.05 compared to the transcriptome-wide background. The online tools Goatods (https://github corn/tanghaibao/Goatools) and KOBAS (http://kobas.cbi.pku.edu.cn/), respectively carried out GO functional enrichment and KEGG pathway analysis.

### 2.16 Brain regions analyses

To further determine the similarity between brain organoid samples cultured for 35 days in AIOMP and CSC and brain regions from fetal to adult stages, the FPKM expression values of 11 time points of fetal development and 16 different brain regions in the Allen BrainSpan human transcriptome dataset (http://www.brainspan.org/static/download.html) were downloaded for correlation analysis. Similarly, in terms of protein expression levels, the proteomic experimental data of 8 PCW human fetal cerebral cortex parts published by Sofia Melliou et al. were also used for similarity analysis of organoids. Firstly, we filter the FPKM values in BrainSpan and organoid data by gene ID for differential analysis, and then Pearson test was used to account for similarity of expression. Finally, Pearson's correlation coefficients between brain organoid samples and brain regions from fetal to adult stages were shown in a heatmap.

### 2.17 Detection of neurotransmitters

Neurotransmitters contents were detected by MetWare (http://www.metware.cn/) based on the AB Sciex QTRAP6500 LC-MS/MS platform. Briefly, the hBOs generated by AIOMP (n = 3, biological replicates = 3) and CSC groups (n = 3, biological replicates = 3) were collected at D100 for neurotransmitters analysis. Subsequently, all organoids underwent three washes with 1 × DPBS followed by preservation in liquid nitrogen and dry ice transportation. The organoid sample were thawed on ice,100 μL of ultrapure water extract (containing protease inhibitors, PMSF and EDTA) was added to resuspend the cell pellet. Divide 50 μL cell suspension and add 200 μL of methanol (precooled at −20 °C)and vortexed for 2 min under the condition of 2500 r/min.The sample was frozen in liquid nitrogen for 5min,removed on ice for 5 min, after that, the sample was vortexed for 2 min.The previous step was repeated for 3 times.The sample was centrifuged at 12000 r/min for 10 min at 4 °C. Take 200 μL of supernatant into a new centrifuge tube and place the supernatant in −20 °C refrigerator for 30 min.Then the supernatant was centrifuged at 12000 r/min for 10 min at 4 °C. After centrifugation, transfer 180 μL of supernatant through Protein Precipitation Plate for further LC-MS analysis.The left 50 μL cell suspension was frozen and thawed for 3 times, centrifuged at 12,000 r/min for 10 min, and the supernatant was taken to determine the protein concentration by BCA Protein Assay kit. The sample extracts were analyzed using an LC-ESI-MS/MS system (UPLC, ExionLC AD, https://sciex.com.cn/;MS,QTRAPR 6500+System, https://sciex.com/).The analytical conditions were as follows, HPLC:column, Waters ACQUITY UPLCHSS T3 C18(100 mm × 2.1 mm i.d.,1.8 μm); solvent system, water with 0.1 %formic acid (A), acetonitrile with 0.1 % formic acid (B); The gradient was started at 5 % B(0 min),increased to 95 % B(0–8 min), 95 % B (8–9.5 min), finaly ramped back to 5 % B (9.6–12 min); flow rate, 0.35 mL/min; temperature, 40 °C; injection volume: 2 μL. AB 6500+QTRAPR LC-MS/MS System, equipped with an ESI Turbo Ion-Spray interface, operating in both positive and negative ion modes and controlled by Analyst 1.6 software (AB Sciex). The ESI source operation parameters were as follows:ion source, turbo spray; source temperature 550 °C; ion spray voltage (IS) 5500 V (Positive), −4500V (Negative); curtain gas (CUR) were set at 35.0 psi; DP and CE for individual MRM transitions was done with further DP and CE optimization. A specific set of MRM transitions were monitored for each period according to the neurotransmitters eluted within this period.

### 2.18 MEA assay

The microelectrode arrays of the 24-well plate (Axion Biosystems, M384-tMEA-24W, 16 electrodes, 1.1 mm × 1.1 mm recording area, 350 μm spacing) were incubated at 37 °C with 5 % CO_2_ for organoid adhesion to the electrodes. Subsequently, 50–100 μL of media was gently added to each well. The organoids were cultured statically in the MEA plate at 37 °C with 5 % CO_2_ until attachment, followed by transfer to the Maestro pro MEA system. Electrical activities were recorded and analyzed using the AxIS Navigator software based on the manufacturer's Spontaneous Neural Configuration. The Neural Metric Tool was utilized to plot electrode array activity. Spike detection threshold was set at six standard deviations using an adaptive threshold crossing method, with spike bursts identified based on an ISI threshold requiring a minimum of five spikes within a maximum ISI of 100 ms.

### 2.19 Statistical analysis

All data were analyzed using GraphPad Prism version 8.0.2 for windows system. Statistical analysis of data was expressed as Means ± SEM. Statistical analysis between two columns was performed using a two-tailed unpaired Student's t-test, whereas data containing more than two experimental groups were analyzed with one-way ANOVA followed by Tukey's multiple comparisons test. In all the analyses, group differences were considered statistically significant as p < 0.05. Sample sizes were indicated in the Figure legends.

## 3 Results

### 3.1 AIOMP simplifies the hBOs production procedure

**Fig. S1:**
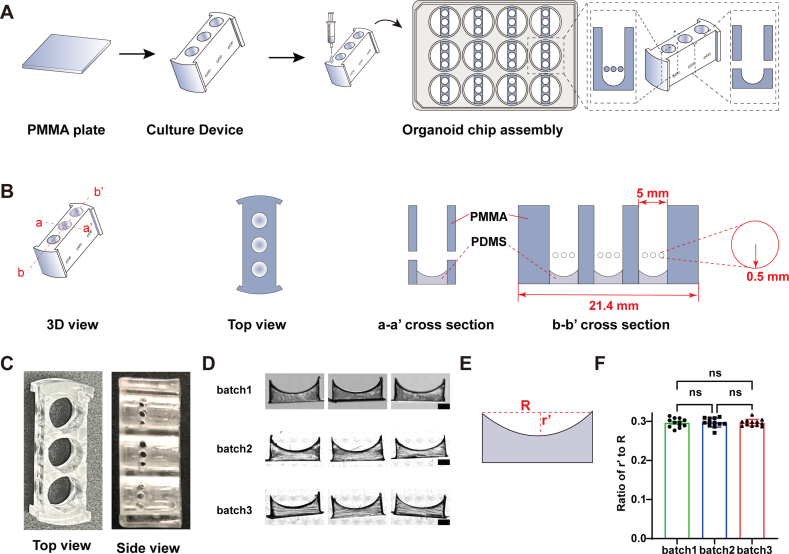
Design and characterization of AIOMP. (A) Schematic diagram of the fabrication process for the AIOMP. (B) Illustration of the 3D view, top view, and cross view for organoid cultivation chamber of the AIOMP and dimensions of each part. (C) Photograph of AIOMP and cultivation chamber. (D-F) Representative bright-field images and quantification analysis of the U-shape bottom in the cultivation chamber of AIOMP (Scale bar, 1 mm; n = 12).

The CSC approach for hBOs formation requires multiple culture devices, generally including a 96-well U-shaped low-adhesion plate for embryonic body formation, a 24-well flat-bottom low-adhesion plate for germ layer differentiation and neural induction, and a 6-well flat-bottom low-adhesion plate for Matrigel encapsulating culture and suspension culture (Fig. 1A). Therefore, the transfer of individual organoids among different devices is requested during the culture procedure. It is laborious and requires a lot of professional hands-on skills. Additionally, the transfer process may cause mechanical damage to the organoids and increase the risk of contamination. The AIOMP simplifies the hBOs culture procedure and integrates all the operational steps on a single millifluidic plate. The AIOMP is built on a standard 12-well plate (Fig. 1B), with each well containing an embedded millifluidic construct that further separates a well into three distinct fluidic regions, including two semicircle medium reservoir regions at the sides and one millifluidic region at the middle (Fig. 1C). The millifluidic construct further consists of 3 separated cylindrical microchambers (5 mm in diameter and 8 mm in height) with each microchamber providing an physically independent culture environment for a single brain organoid culture (Fig. S1B), which avoids organoid collision and fusion frequently occurring in the CSC [30]. Specifically, the microchambers contain a PDMS-based U-shaped low-adhesion substrate for aggregating 3 × 10^3^ hESCs into a single embryonic body in 12 h. Additionally, every microchamber is designed with 3 tunnels fluidically connecting to two side reservoirs for bidirectional perfusion culture at the last stage of the hBOs differentiation (Fig. S1A–C). The perfusion culture is conducted by placing the AIOMP on a rocker shaker. The perfusion culture explores gravitational flow and can be easily scale-up without adding any active pump. Five stages of differentiation are conducted on the AIOMP without the need for organoid transfer.

### 3.2 AIOMP enhances the homogeneity and reproducibility of hBOs formation

To examine the homogeneity of hBOs, we performed the size analysis of hBOs at the different culture stages. The results showed that the average diameter of hBOs increased over time (Fig. 1D). On day 35, the average diameters were 3.15 ± 0.34 mm and 3.09 ± 0.62 for the AIOMP and CSC groups, respectively, and the ratio of length to width of the hBOs were 1.15 ± 0.02 and 1.32 ± 0.20 for the AIOMP and CSC groups, respectively (Fig. 1E and F). The typical images of the cultured hBOs (D35) in an AIOMP and a 6-well plate were shown in Fig. 1G and H, respectively. The variability analysis indicated that the coefficient of variation (CV) of the average diameter and the ratio of length to width for the AIOMP were 0.06 ± 0.02 and 0.03 ± 0.01, respectively, while those for the CSC were 0.16 ± 0.02 and 0.07 ± 0.02, respectively. We performed the morphological analysis of neural rosettes, recognized as a typical structural marker of hBOs morphogenesis during neurodevelopment. The results showed that 70 % of hBOs in the AIOMP took a morphological score larger than 0.5, while only 35 % of hBOs in the CSC took the same score (Fig. 1K and L). These results indicate that the AIOMP improves the homogeneity of hBOs as well as the efficiency of neural morphogenesis.

### 3.3 AIOMP provides a well-defined biophysical microenvironment

**Fig. 2:**
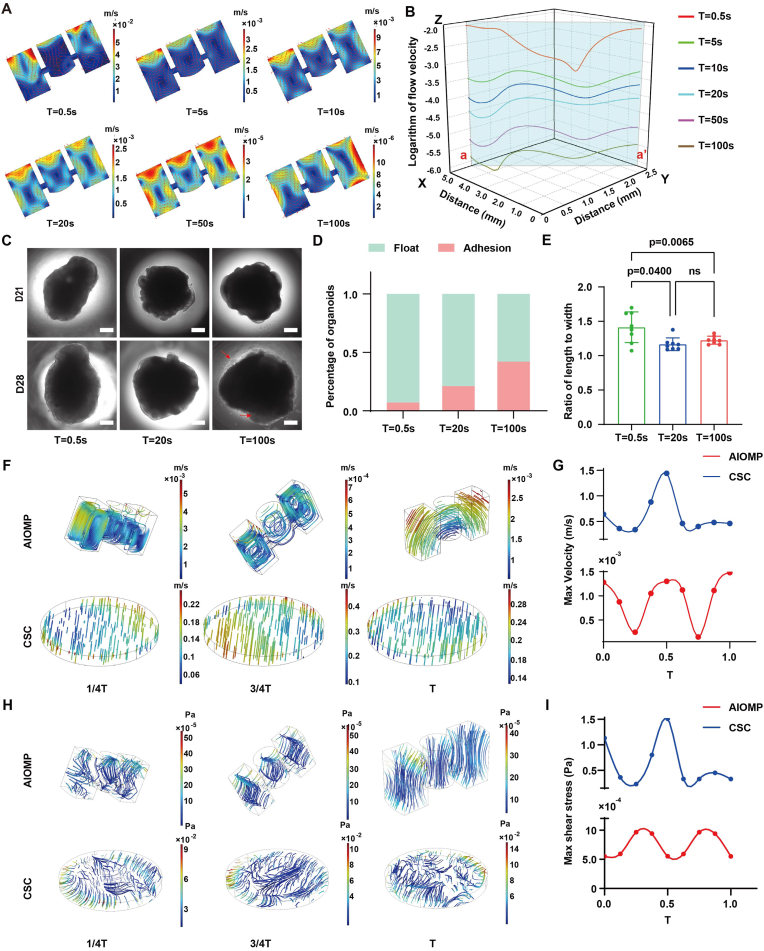
Numerical simulations and condition optimization for dynamic culture. (A) Computational fluid dynamic analysis of fluid velocity for the AIOMP under different rotation cycle conditions (T = 0.5 s, 5 s, 10 s, 20 s, 50 s, and 100 s). (B) The fluid dynamic analysis and statistical analysis of flow velocity under the condition of an inclination angle of 0°. (C) Bright-field images of hBOs under different rotation conditions during differentiation (D21 and D28) (Scale bar = 500 μm). (D) The percentages of growth state analysis of hBOs (n = 12). (E) The ratio of length to width of the hBOs in different rotation conditions (T = 0.5 s, 20 s, and 100 s). (F) Distribution analysis results of the velocity under different cycle conditions in organoid culture chamber and 6-well plate (n = 8). (G) Quantitative analysis of the velocity simulation results in organoid culture chamber of AIOMP and 6-well plate within a single cycle. (H) Distribution analysis results of shear stress under different cycle conditions in organoid culture chamber of AIOMP and 6-well plate. (I) Quantitative analysis of shear stress simulation results in organoid culture chamber and 6-well plate within a single cycle.

We performed computational fluid dynamics (CFD) simulation using COMSOL Multiphysics to optimize the biophysical microenvironment of the AIOMP culture. Specifically, we interrogated microflow dynamics under varied rocking periods (T = 0.5, 5, 10, 20, 50, and 100 s) with a fixed tilt angle of 25°. The simulation revealed that the velocity in the culture chamber was negatively correlated to the rocking period (Fig. 2B). When the rocking period was 5 s or more, steady vortex flow was formed in the microchamber and enhanced culture medium exchange (Fig. 2A). The simulation further demonstrated that the velocity were ranged from 1.31 × 10^−5^ m/s (T = 100 s) to 6.00 × 10^−2^ m/s (T = 0.5 s). The bright-field image analysis of the hBOs culture on day 28 further illustrated that a low velocity (T = 100 s) resulted in a high proportion of organoid adhesion (42 %) to the substrate and cell migration from the hBOs (Fig. 2C and D). Conversely, a large velocity (T = 0.5 s) resulted in deformed morphogenesis of hBOs with a high axial ratio (2.09 ± 0.20) (Fig. 2C and E). Therefore, we finally selected a rocking period of 20 s to avoid these adverse effects in the subsequent experiments.

**Fig. S2:**
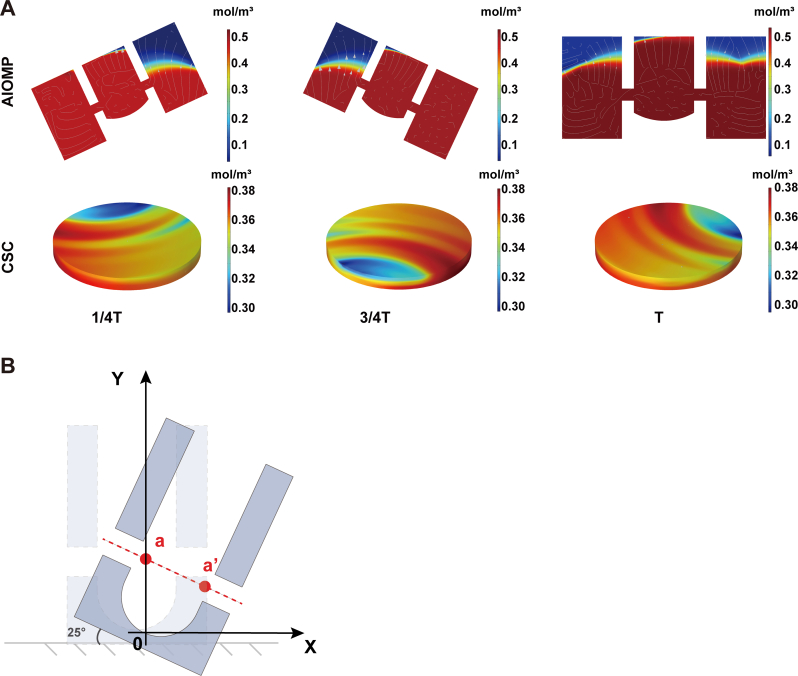
**Diffusion analysis for the AIOMP and CSC groups.** (A) Distribution analysis results of diffusion under different cycle conditions in organoid culture chamber of AIOMP and 6-well plate. (B) Spatial schematic diagram of flow velocity simulation.

Additionally, we compared the AIOMP culture (T = 20 s) with the CSC in their fluid dynamic environment at the last stage of hBOs differentiation using numerical simulation. The convection and diffusion analysis illustrated that the AIOMP culture provided a more uniform culture medium exchange than the CSC during one cycle (Fig. S2). The streamline analysis further indicated a stable vortex existing in the microchamber that may enable an efficient medium exchange around the hBOs (Fig. 2F). The maximum velocity in the AIOMP was 4.14 × 10^−4^ m/s, which was three magnitudes less than that in the CSC (0.61 m/s) (Fig. 2G). Moreover, shear stress analysis indicated that the maximum shear stress was calculated as 1 × 10^−3^ Pa for the AIOMP and 1.5 Pa for the CSC (Fig. 2H and I), revealing that the AIOMP culture provides a biocompatible fluidic environment to neural parenchymal cells.

### 3.4 AIOMP enhances early-stage corticogenesis in the hBOs

**Fig. 3:**
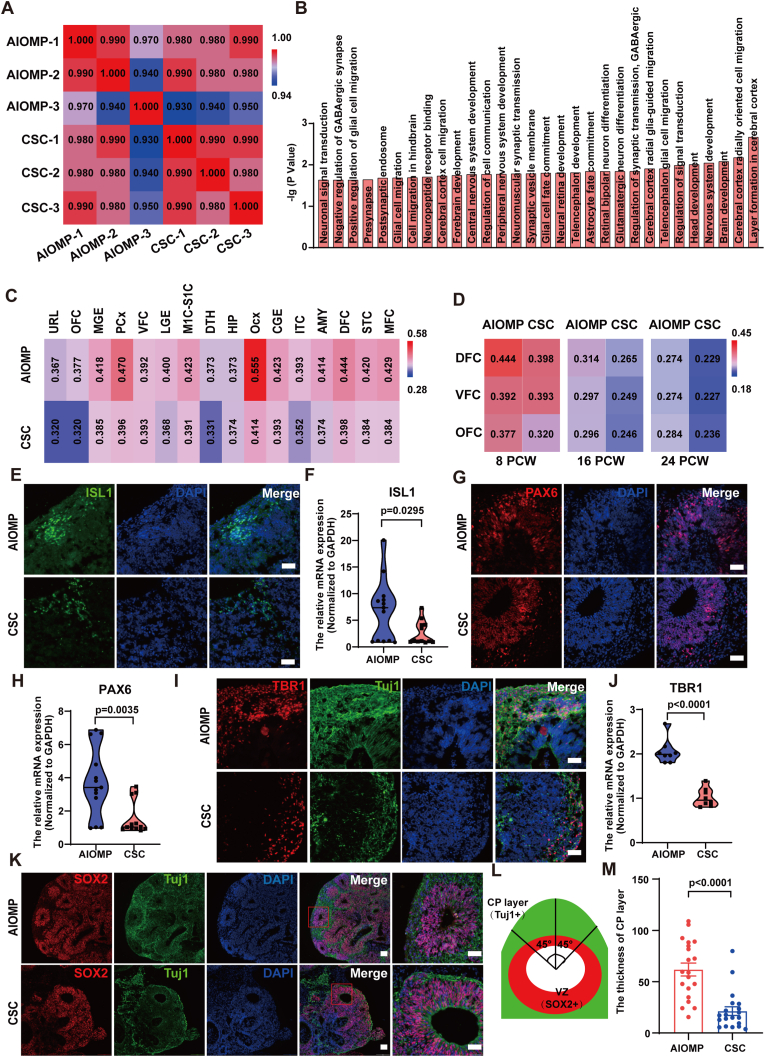
AIOMP enhances corticogenesis and maturation in hBOs. (A) Pearson correlation matrix for transcriptome-wide profiles of organoids in the AIOMP and CSC group. Pearson's correlation coefficient (PCC) values are indicated in each box. (B) The top 30 enriched Gene Ontology (GO) neurodevelopment-related terms of upregulated genes in the AIOMP/CSC (shown in terms of p values). (C–D). Heatmaps of Pearson's correlation analysis of RNA-sequencing datasets of the AIOMP and CSC organoids for comparison with published Allen Brain Span human transcriptome dataset of human frontal regions across different stages and (C) different regions of the brain at postnatal stages. Pearson's Correlation Coefficient (PCC) values are displayed in a heatmap. (E, F) The expression of hindbrain marker (ISL1) was identified by immunofluorescence staining, and the mRNA of *ISL1* (F) was quantified by RT-qPCR in the AIOMP and CSC group (Scale bar = 100 μm, independent replicate = 3). (G, H) The expression of forebrain marker (PAX6) was identified by immunofluorescence staining, and the mRNA of *PAX6* (I) was quantified by RT-qPCR in the AIOMP and CSC group (Scale bar = 100 μm, independent replicate = 3). (I) Immunostaining for markers of deep-layer neuron markers (TBR1), and immature neurons (Tuj1) in hBOs by AIOMP or CSC (F) at D35. (J) Transcripts for *TBR1* were examined by RT-qPCR of hBOs in the AIOMP and CSC group at D35 (Scale bar = 100 μm, independent replicate = 3). (K, L) Immunofluorescence image and schematic representation of ventricular zone (VZ, SOX2^+^) and cortical plate layer (CP, Tuj1^+^) measurement in cortical structures and (M) plot for relative CP thickness in the day 35 hBOs (Scale bar = 100 μm, independent replicate = 3). The relative levels of genes were normalized to GAPDH. Data represent the mean ± SEM.

**Fig. S4:**
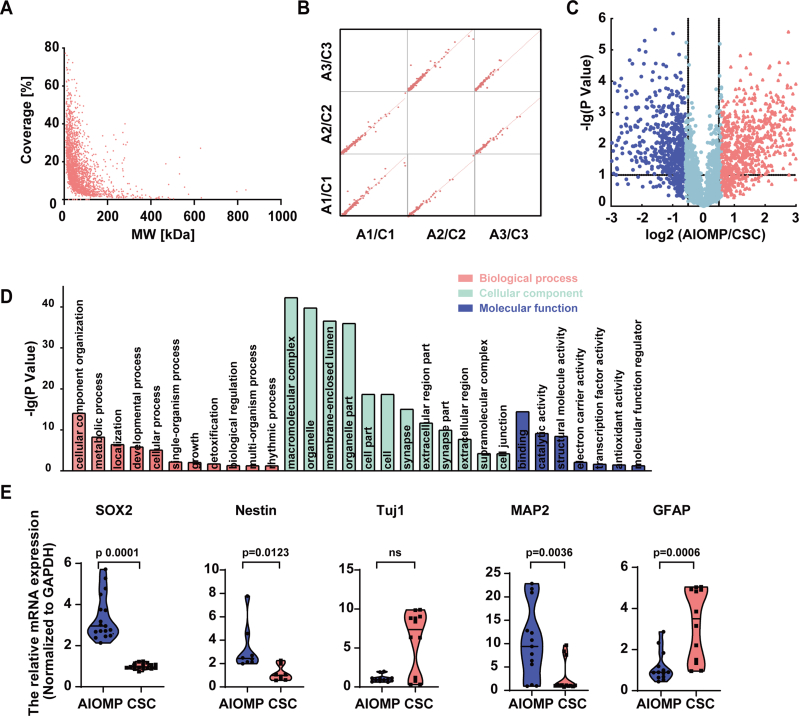
AIOMP enhances neuron associated transcriptional gene expression in organoids. (A) The violin box plot indicates the overall dispersion of expression. The horizontal axis abscissa in the figure represents different samples, and vertical axis represents the logarithmic value of the sample expression although FPKM. (B) Heat map analysis of all gene expression levels. (C) PCA cluster analysis of transcriptome gene expression. (D) Volcano plot analysis of transcriptome DEGs. Red represents upregulated genes and green represents downregulated genes, and GO annotation information of DEGs, according to the distribution of upregulated proteins of BP (E) CC (F) and MF (G) in GO terms of level 2. (H and I) RT-qPCR showing expression of forebrain (*FOXG1*) and hindbrain (*PAX2*) genes in the cerebral organoids in AIOMP and CSC groups at D35.

**Fig. S5:**
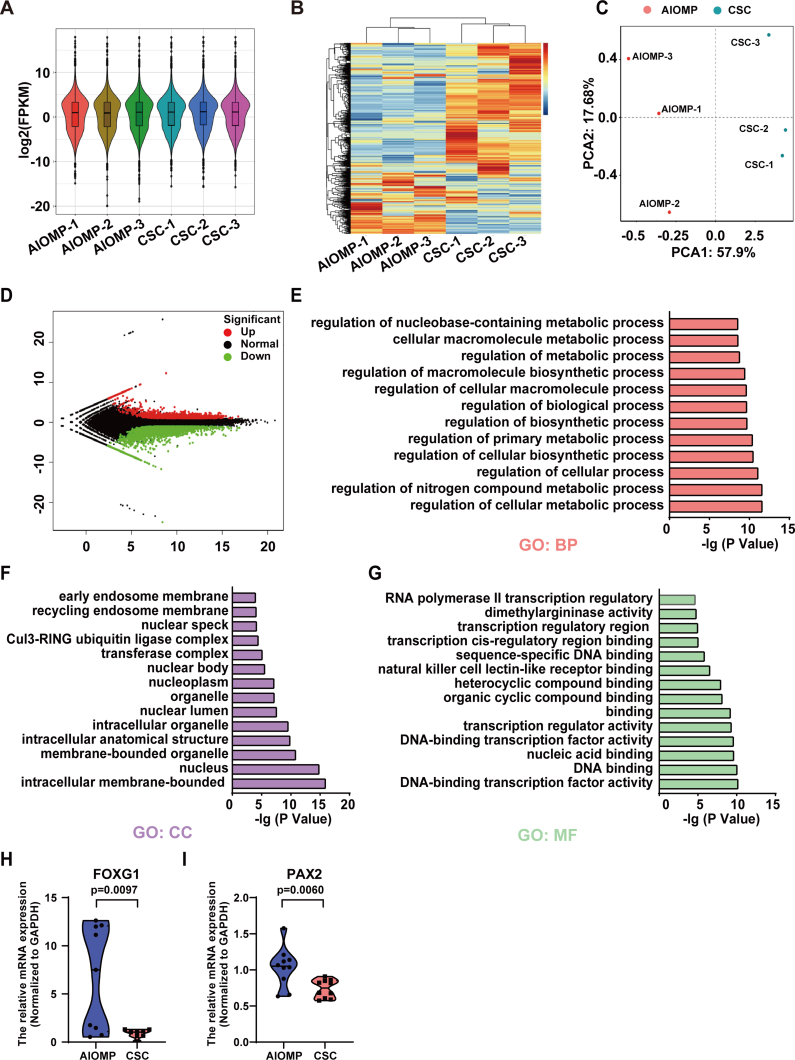
Profiling of LFQ proteomics in brain organoids generated in AIOMP and CSC. (A) The coverage analysis in globe proteomics. (B) Pearson correlation coefficient test for triplicate experiments. (C) Volcano plot of differential proteins. (D) Functional analysis in GO enrichment of different expressed proteins according to Biological Processes (BP), Cellular Components (CC), and Molecular Functions (MF). (E) RT-qPCR showing expression of neural progenitor (*SOX2*, *Nestin*), neuron (*Tuj1*, *MAP2*), and astrocyte (*GFAP*) genes in the brain organoids in AIOMP and CSC groups at D35. Data represent the mean ± SEM.

We performed global transcriptome RNA-sequencing analysis on three batches of the hBOs generated by the AIOMP and the CSC on Day 35. Pearson's correlation coefficient indicated a reliable reproducibility for three repeated experiments (Fig. 3A). Global mRNA expression levels were displayed by heatmap (Fig. S4B) and analyzed by PCA (Principal Component Analysis) clustering method (Fig. S4C). Compared to the CSC organoids, the differentially expressed genes (DEGs) demonstrated that 3114 genes were downregulated and 1324 genes were upregulated in the AIOMP organoids (Fig. S5D). Gene Ontology (GO) analysis further showed that the neurodevelopment-related genes, especially the genes related to corticogenesis (Fig. 3B), BP, CC, and MF were significantly upregulated in the AIOMP organoids (Fig. S5E–G).

We examined the neurodevelopmental correlation between the hBOs and human fetal brains at the gestational weeks. Specifically, the transcriptional profiles of the human fetal brains were retrieved from 16 different parts of the human brain cortex from 8 post conception weeks (PCW) (Fig. 3C). Pearson's correlation coefficient indicated that hBOs in both culture groups were more closely related to the brain development at 8 PCW (Fig. 3D). Particularly, the hBOs in both groups were highly correlated with occipital neocortex (Ocx) development in the human brain. Notably, the AIOMP organoids demonstrated a higher relevance with the fetal brain in all the regions than the CSC organoids and were especially highly correlated with cortical regions (DFC, dorsolateral prefrontal cortex; VFC, ventrolateral prefrontal cortex; OFC, orbital frontal cortex).

Furthermore, we performed immunofluorescence analysis to verify the RNA sequencing. The results showed that the both the AIOMP and CSC organoids expressed forebrain (PAX6) and hindbrain (ISL1) developmental markers (Fig. 3E). RT-qPCR analysis further confirmed that both forebrain-specific genes (*PAX6*, *FOXG1*) and hindbrain-specific genes (*ISL1*, *PAX2*) were significantly upregulated in the AIOMP organoids compared to those in the CSC organoids (Fig. 3F and H, Figure S5H and I). Moreover, we examined the emergence of the cortical plate (CP) and ventricular zone (VZ), a pivotal neurodevelopment structure in the hBOs. Immunofluorescence analysis showed that cortical newborn neurons distributed in the outer layer of hBOs (Fig. 3I), which was consistent with the locations of newborn neurons in human fetal brain [45,46]. RT-qPCR analysis further showed that the gene expression of TBR1, a T-box transcription factor expressed in the cerebral cortex, was significantly upregulated in the AIOMP compared with the CSC (Fig. 3J). In addition, we quantified the structures of CP (Tuj1^+^) and VZ (SOX2^+^) in the hBOs. We found that the CP region in the AIOMP organoids was thicker than that in the CSC organoids, which revealed that the AIOMP culture significantly promoted early-stage corticogenesis in the hBOs (Fig. 3K and L).

### 3.5 AIOMP improves neurogenesis in the hBOs

**Fig. 4:**
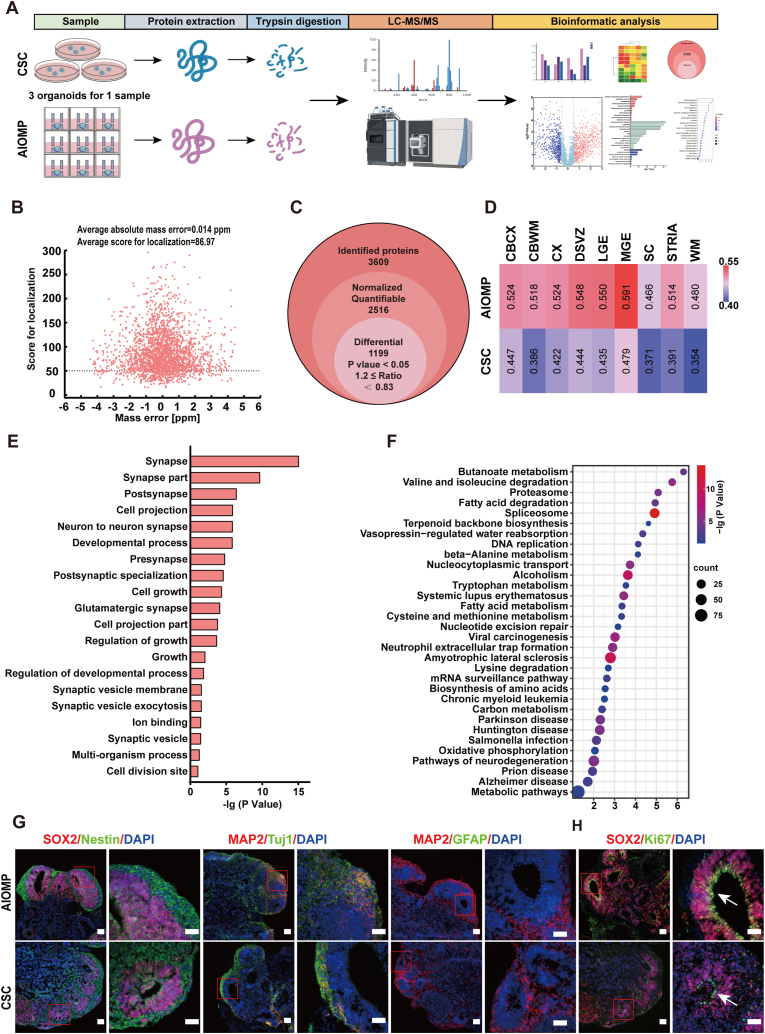
**AIOMP improves neurogenesis in the hBOs.** (A) Schematic representation of label-free quantitative proteomics of the organoids generated in the AIOMP and CSC. (B) Distribution of the peptides mass error (ppm) based on *m*/*z* of 2516 identified peptides. (C) Venn diagram showing the total numbers of proteins identified in the AIOMP/CSC organoids. (D) Volcano plot of differential protein quantification for proteomics. Red represents upregulated proteins and Purple represents downregulated proteins. (E) Top 20 neurodevelopment-related enriched Gene Ontology (GO) terms of upregulated proteins in the AIOMP/CSC organoids and ordered by p value. (F) Bubble plot showing top enriched items for KEGG pathway analysis in differential proteins. (E) The top 30 enriched gene ontology (GO) terms of upregulated genes in the AIOMP versus the CSC group (shown in terms of p values) (F) Immunostaining of the marker of neural stem cell (SOX2 and Nestin), neuron (Tuj1 and MAP2), and astrocyte (GFAP) in the AIOMP and CSC groups at D35 (Scale bar = 100 μm). (G) Immunostaining for the proliferation marker Ki67 and the progenitor marker SOX2 in the AIOMP and CSC group at D35 (Scale bar = 100 μm). (n = 3). (For interpretation of the references to colour in this figure legend, the reader is referred to the Web version of this article.)

We conducted a comprehensive comparative large-scale label-free quantitative proteomics analysis of the AIOMP and CSC organoids on Day 35. As shown in Fig. 4A, equal amounts of the whole protein were extracted from the hBOs, digested using trypsin, detected by LC-MS/MS (Q Exactive HF-X, Thermo Fisher Scientific), and ultimately analyzed by bioinformatic tools. Specifically, the overall average absolute peptide mass error was 0.014 ppm and the average peptide score for localization was 86.97 (Fig. 4B). The reproducibility analysis of Pearson's correlation coefficient for three repeated experiments was shown in Fig. S4B. Then, using the LFQ algorithm in MaxQuant (V1.6.6), a total of 3609 proteins with localization probes ≥0.75 were detected in our experiments under different conditions (AIOMP/CSC). The search result data was filtered using Perseus (1.6.15.0), 2516 proteins were quantifiable (Fig. 4C). A comparison of differentially expressed proteins (DEPs) showed that 1088 proteins were upregulated (quantitative ratio ≥1.5, p value < 0.05), and 875 proteins were significantly downregulated (quantitative ratio ≤0.67, p value < 0.05) in the AIOMP compared to the CSC (Fig. S5C).

We examined the neurodevelopmental relevance of the hBOs with human fetal brains at the protein expression level. Human fetal brain proteomics dataset was retrieved from Sofia Melliou parts of the human brain cortex at 8 PCW for correlation analysis [47]. Pearson's correlation coefficient indicated that the AIOMP-generated hBOs showed a higher similarity to the early-stage fetal brain development than the CSC hBOs (Fig. 4D).

Furthermore, functional annotation and Gene Ontology (GO) analyses were carried out using OmicsBox (2.0.24X) [48]. 1190 DEPs were classified according to their biological processes (BP), cellular components (CC), and molecular functions (MF) (Fig. S5E–G) (Table S2). From the biological process perspective, the full set of DEPs were associated with development process and metabolic processes. To gain further insight into the functional roles of the identified DEPs, the automated analysis tool, DAVID Bioinformatics Resources, was used to perform enrichment analysis of the DEPs. In order to investigate the development of organoids, GO-BP analysis was mainly concerned. We found that the protein abundance of hBOs in the AIOMP was higher than the CSC. And these upregulated proteins were mainly enriched in cellular component organization or biogenesis (p = 8.77E-15), metabolic process (p = 5.54E-09), localization (p = 3.69E-07), developmental process (p = 1.46E-06), cellular process (p = 8.30E-06) and growth (p = 0.0089). There was a large number of enriched proteins in the macromolecular complex (p = 5.25E-43), organelle (p = 1.75E-40), synapse (p = 1.75E-40), synapse part (p = 1.17E-10) and cell junction (p = 6.59E-05). The MFs of DEPs included the binding (p = 3.60E-15), structural molecule activity (p = 3.58E-09), electron carrier activity (p = 0.0079) and transcription factor activity (p = 0.023) (Fig. 4E). This dataset implied that the upregulated proteins in the AIOMP were mainly enriched in synapse formation and neurogenesis. We next performed Kyoto Encyclopedia of Genes and Genomes (KEGG) metabolism-related protein pathway enrichment analysis, KEGG metabolic pathway analysis showed that the most enriched upregulated proteins categories were the Spliceosome (p = 2.19E-14), followed by Amyotrophic lateral sclerosis (p = 1.43E-10), Viral carcinogenesis (p = 2.60E-07), Pathways of neurodegeneration (p = 9.92E-06) and Huntington disease (p = 1.53E-05) (Fig. 4F).

We further performed immunofluorescence and RT-qPCR analysis of the hBOs (D35) to verify the results of proteomics (Fig. 4G, H and S4E). Immunofluorescence analysis showed that the hBOs in both groups expressed neural stem cell markers (SOX2 and Nestin) and neuronal markers (Tuj1 and MAP2). However, glial cell marker (GFAP) was not detected in these hBOs. RT-qPCR analysis further indicated that the neural stem cell markers (*SOX2* and *Nestin*) were significantly upregulated by 3.36 and 2.88 folds in the AIOMP compared to the CSC. Simultaneously, the maturated neuronal marker (*MAP2*) was upregulated by 3.32 folds in the AIOMP compared to the CSC. However, the expression of an immature neuronal marker (*Tuj1*) didn't show a significant difference. Although glial cell marker (*GFAP*) was detected at the mRNA level, it was significantly downregulated in the AIOMP-generated hBOs compared to the CSC. All these results indicated that the AIOMP effectively promoted the differentiation of hBOs into neural lineages.

### 3.6 AIOMP promotes the long-term cultivation and functional maturation of hBOs

**Fig. 5:**
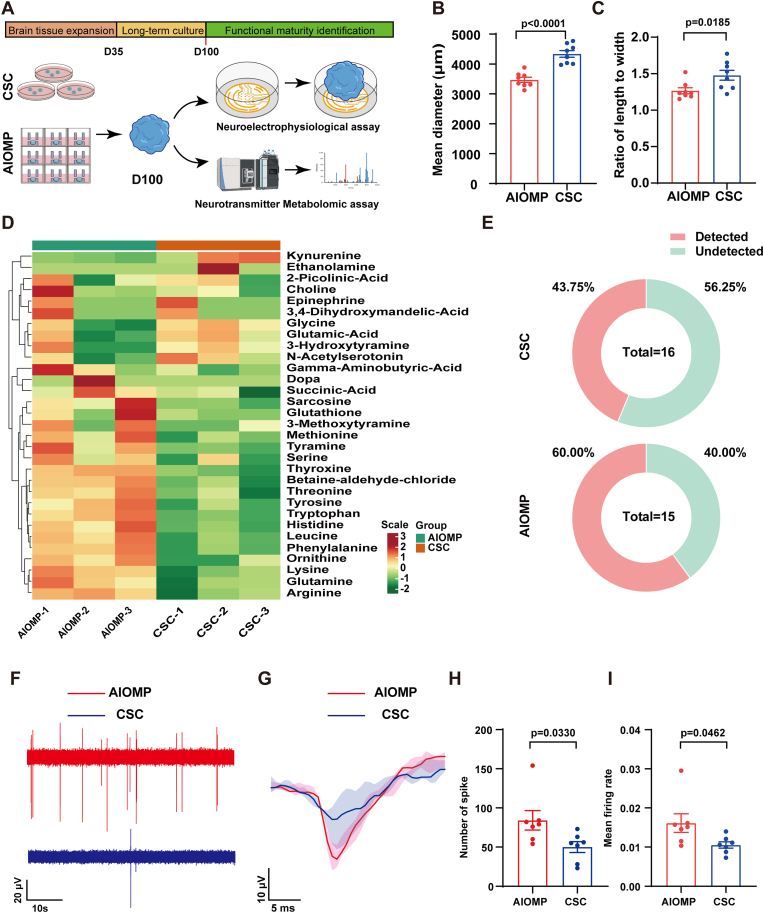
**AIOMP facilitates the long term cultivation and functional maturation of hBOs.** (A) Schematic illustration of electrophysiological activity and neurotransmitter detection in brain organoids (D100) in the AIOMP and CSC groups. (B, C) Diameter and length-to-width ratio of brain organoids in the AIOMP and CSC groups (n = 12). (D) Heatmap comparing the relative synthesis levels of 31 neurotransmitters in the AIOMP and CSC groups. (E) Donut charts showing the proportions of detected and undetected electrical signals in the AIOMP and CSC groups. (F) Representative image of action potentials in brain organoids in the AIOMP and CSC groups. (G) Typical electrographic features in brain organoids in the AIOMP and CSC groups. (H) Statistical graph of number of spikes in 5 min. (I) Statistical graph of mean firing rates (Hz) in brain organoids in the AIOMP and CSC groups. Data represent the mean ± SEM. (n ≥ 7).

**Fig. S6:**
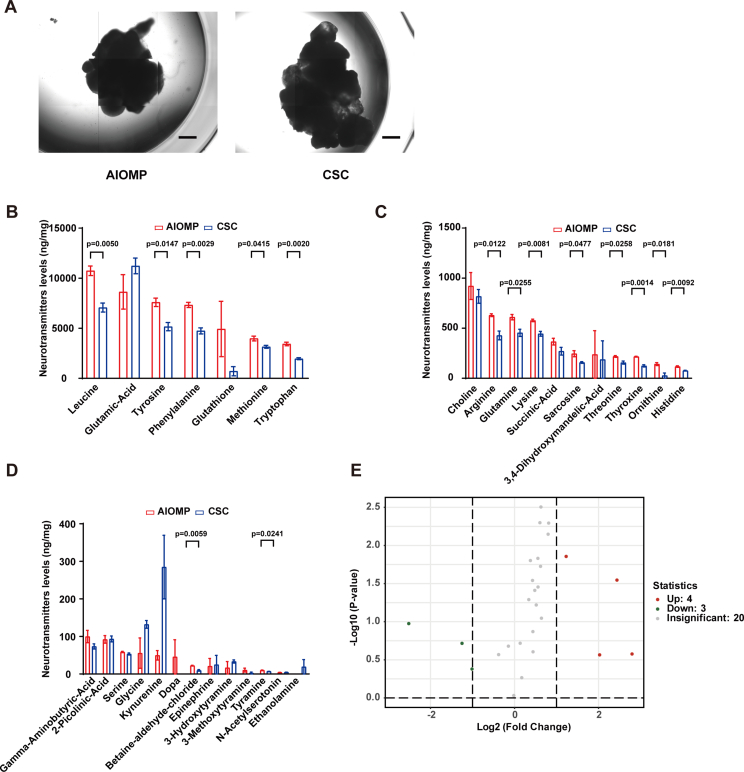
The long-term cultivation of brain-like organs and neurotransmitter levels produced in AIOMP and CSC. (A) Bright-field images of brain organoids at D100 (Scale bar, 1 mm). (B-D) Statistical graphs of neurotransmitter synthesis levels. (n = 3) (E) Volcano plot of differential neurotransmitters. (F) The radar plot of differential neurotransmitters. Data represent the mean ± SEM.

To further evaluate the impact of AIOMP on the long-term cultivation and electrophysiological functions of hBOs, we cultured hBOs in both AIOMP and CSC for 100 days, subsequently conducting a comprehensive analysis that included morphological assessment, neurotransmitter metabolomics, and electrophysiological functionality (Fig. 5A). The typical images of the cultured hBOs (D100) in AIOMP and CSC were shown in Fig. S6. Morphological analysis at D100 revealed significant differences in the mean diameter and length-to-width ratio between organoids in the AIOMP and CSC groups. Specifically, the AIOMP group exhibited a mean diameter of 3508.74 ± 82.36 μm and a length-to-width ratio of 1.26 ± 0.03, which were notably smaller and more uniform than those in the CSC group, measured at 4363.46 ± 127.57 μm and 1.42 ± 0.11 respectively (p = 0.0001 for diameter and p = 0.0185 for length-to-width ratio, Fig. 5B and C). This indicates that AIOMP cultures yield more uniform organoids, consistent with previous findings on D35 hBOs. Moreover, neurotransmitter-targeted metabolomics analysis of organoids from both groups at D100 identified 31 quantifiable neurotransmitters, with the AIOMP group showing higher levels of 15 neurotransmitters compared to the CSC group (Fig. 5D). Notably, 7 neurotransmitters in the AIOMP group, including leucine, glutamic acid, tyrosine, phenylalanine, glutathione, methionine, and tryptophan, exceeded a protein content of 1000 ng/mg (Fig. S6B–D). Comparative analysis indicated that 4 neurotransmitters were upregulated (quantitative ratio ≥1.5, p value < 0.05) and 3 neurotransmitters were significantly downregulated (quantitative ratio ≤0.67, p value < 0.05) in AIOMP compared to CSC (Fig. S6E), suggesting an enhanced neurotransmitter synthesis capacity in AIOMP-cultured organoids. Additionally, we employed the MEA method to assess the electrophysiological activity of hBOs cultured for 100 days in both AIOMP and CSC environments. The results indicated that a significant 60 % of brain organoids in the AIOMP group exhibited electrophysiological signals, compared to only 43.75 % in the CSC group (Fig. 5E). The notable difference between the two groups is visually depicted in Fig. 5F and G, showing the spike traces (1 min) and the waveform of the electrical signal (20 ms). Quantitatively, the AIOMP group demonstrated a significantly higher number of spikes (92.57 ± 16.47 vs. 49.71 ± 7.79, p = 0.0330) and mean firing rates (0.0147 ± 0.0006 Hz vs. 0.0107 ± 0.0005 Hz, p = 0.0462), as depicted in the statistical graphs of Fig. 5H and I. These findings suggest that AIOMP not only supports the long-term cultivation of brain organoids but also effectively promotes their electrophysiological function maturation.

## 4 Discussion

Human brain organoids (hBOs) have emerged as a transformative model for exploring human neurodevelopment and disease. However, the conventional standard culture (CSC) method faces several challenges, including procedural complexity, high variability, and limited functional maturity. Recently, organoid engineering has progressed to meet this challenge through techniques like 3D bioprinting for spatial patterning [44,49], assembloids for modeling region-specific interactions [50,51], and microfluidic organ-on-a-chip platforms for enhanced microenvironmental control [34,40,41,52]. Although these approaches have achieved significant advancements, a critical gap often persists in the comprehensive biological validation of engineered organoids across molecular, cellular, and functional levels [53]. Many engineering-focused studies prioritize device innovation or morphological analysis but lack comprehensive multi-omic profiling and functional electrophysiological validation, which are crucial for confirming the biological relevance and accuracy of the model. Our study directly addresses this issue by introducing an AIOMP that not only streamlines and standardizes the production of hBOs but also provides solid multi-scale biological characterization.

Compared to existing engineering strategies, the AIOMP offers a unique combination of simplicity, scalability, and control. While microfluidic devices with closed channels enable precise fluid dynamics but can physically restrict organoid growth, leading to deformation [53], the AIOMP's open-channel, millifluidic design provides an unrestricted space for unhindered organoid morphogenesis while enabling controlled perfusion. Unlike systems that depend on complex syringe pumps [39,40,54,55], the AIOMP uses a simple rocking platform to generate gravity-driven flow, making it easily scalable and accessible. This design integrates all culture steps—from embryonic body formation to long-term maturation—into a single device, eliminating the laborious and damaging transfers inherent in CSC and other engineered platforms [56]. Our results show that this integrated method produces better uniformity, as indicated by the significantly lower coefficient of variation in diameter (CV: 0.06 ± 0.02 vs. 0.16 ± 0.02) and axial ratio of AIOMP-generated hBOs. This streamlined, all-in-one workflow offers a major practical benefit for labs seeking a consistent and easy-to-use platform. The addition of real-time monitoring features further helps researchers evaluate organoid responses to various stimuli, supporting a deeper understanding of their behavior and applications. As we continue exploring this platform, we expect the AIOMP will not only fill current gaps in organoid research but also establish a new benchmark for engineering and applying organoid systems across different biomedical fields.

A key innovation of the AIOMP is its ability to impart a biomechanically relevant microenvironment. The CFD simulations confirmed that the AIOMP employs a millifluidic system, in which a laminar flow with a uniform velocity field and low shear stress is formed. This is achieved by a gravity-driven rocking motion (25° angle, 20 s cycle), which generates a steady vortex flow within each microchamber. Computational fluid dynamics revealed a maximum velocity of 4.14 × 10^−4^ m/s and a maximum shear stress of 1 × 10^−3^ Pa in the AIOMP, values that fall within the physiological range of human cerebrospinal fluid (CSF velocity: 10^−4^ to10^−3^ m/s; shear stress: ∼10^−3^ Pa) [57]. Thus, hBOs in the AIOMP experience a biomechanical microenvironment that simulates key hemodynamic features of CSF, ensuring uniform organoid formation. This bio-inspired dynamic conditioning likely contributes to the increased maturation seen in our study, setting the AIOMP apart from static culture systems and even some dynamic systems that do not replicate this specific physiological feature. The stable, mass-transfer-efficient microenvironment prevents organoid adhesion and cell migration at low flow rates (T = 100s) and avoids deformation at high flow rates (T = 0.5s), emphasizing the importance of optimized engineering parameters for healthy organoid development. Furthermore, precise control over the microenvironment enhances nutrient and oxygen delivery, both essential for cellular metabolism and growth. This optimization not only maintains cell viability but also promotes the expression of key markers linked to organoid maturity. Additionally, the AIOMP's design allows for real-time monitoring and adjustments, enabling researchers to customize conditions based on specific experimental needs. Such flexibility is vital for exploring various biological questions and therapeutic applications, making the AIOMP a versatile tool in organoid research.

A major strength of our work is the rigorous multi-omic validation of the AIOMP-derived hBOs, a level of analysis often missing in purely engineering-focused papers. Our transcriptomic and proteomic analyses at D35 consistently showed that the AIOMP microenvironment actively promotes neurodevelopment. The stronger correlation of AIOMP-hBOs transcriptomes with the human fetal brain at 8 PCW, especially in cortical regions like the dorsolateral prefrontal cortex, indicates a more in vivo-like regional specification. This was further confirmed by proteomics, where AIOMP-hBOs exhibited a higher similarity to the human fetal brain proteome at 8 PCW than CSC-hBOs [47,58]. The upregulation of 1088 proteins in the AIOMP group, which are enriched in critical biological processes such as “developmental process” and “synapse organization,” confirms enhanced neural and cortical development at the molecular level. The coordinated increase in both neural progenitor markers (SOX2, Nestin) and mature neuronal markers (MAP2) suggests that the AIOMP promotes an actively neurogenic niche, rather than simply speeding up a uniform maturation timeline. By going beyond typical morphological evaluations, this multi-omic integration provides a strong molecular basis for the platform's superiority.

Furthermore, we extended our analysis to 100 days to directly address functional maturation, a common critique of short-term organoid studies. The AIOMP allows for long-term cultivation of organoids with more consistent size and shape than their CSC counterparts. The metabolomic analysis showed increased production of 15 neurotransmitters, including important molecules like glutamic acid and tryptophan. Most importantly, microelectrode array (MEA) recordings provided definitive evidence of advanced functional maturation: 60 % of AIOMP-hBOs exhibited spontaneous electrophysiological activity, with significantly higher spike counts and mean firing rates than CSC-hBOs. This combination of metabolomic and neurophysiological data demonstrates that the AIOMP not only improves structural and molecular fidelity but also drives the development of functional neural networks, a key milestone for modeling neurological disorders and drug screening.

## 5 Conclusion

In conclusion, the AIOMP represents a significant advance in brain organoid engineering by combining a simple, reliable platform with comprehensive multi-scale biological validation. Its innovative design addresses major pitfalls of conventional and existing engineered methods by integrating all culture steps, providing a physiologically relevant microenvironment, and producing organoids with enhanced homogeneity, molecular fidelity, and functional capacity. By rigorously coupling engineering innovation with comprehensive multi-omic and electrophysiological analyses, our study sets a new standard for validating bioengineered brain models. We anticipate that the AIOMP will become a valuable tool for reproducible and biologically relevant applications in neurodevelopmental modeling, disease research, and central nervous system drug discovery.

## CRediT authorship contribution statement

**Wen Zhao:** Writing – original draft, Visualization, Software, Methodology, Investigation, Formal analysis, Data curation. **Yu Wang:** Writing – original draft, Visualization, Methodology, Investigation, Formal analysis, Data curation. **Tao Chen:** Validation, Investigation. **Min Shen:** Software, Investigation, Data curation. **Jibo Wang:** Visualization, Software, Methodology, Investigation. **Xuemei Huang:** Validation, Investigation. **Lili Zhu:** Validation, Investigation. **Ting Yu:** Investigation. **Zhentao Zhang:** Writing – review & editing, Supervision, Resources, Investigation. **Yunhuang Yang:** Writing – review & editing, Supervision, Resources. **Maili Liu:** Supervision, Resources. **Dong Wang:** Funding acquisition. **Weihua Huang:** Supervision, Resources. **Rui Hu:** Writing – review & editing, Supervision, Resources, Project administration, Investigation, Funding acquisition, Formal analysis, Conceptualization. **Pu Chen:** Writing – review & editing, Writing – original draft, Validation, Resources, Project administration, Methodology, Investigation, Funding acquisition, Formal analysis, Data curation, Conceptualization.

## Data and materials availability

All data needed to evaluate the conclusions in the paper are present in the paper and/or the Supplementary Materials. The sequencing data produced in this study have been deposited in the NCBI Sequence Read Archive (SRA) under the BioProject accession number PRJNA1022812, and the mass spectrometry proteomics data have been deposited to the ProteomeXchange Consortium via the PRIDE partner repository with the dataset identifier PXD045839.

## Funding

This study was supported by the 10.13039/501100001809National Natural Science Foundation of China (82272173, 32571639, 22374155, 21991080) and Science and Technology Special Fund of Hainan Province (ZDYF2025SHFZ027). The funders had no role in study design, data collection and analysis.

## Declaration of competing interest

P.C. is a founder of, and has an equity interest in: Hefei Ranyin BioTechnologies Co., Ltd., a company that is developing PSC-derived organoids for new drug discovery. P.C.’s interests were viewed and managed in accordance with the conflict-of-interest policies.

## Figures and Tables

**Fig. S3:**
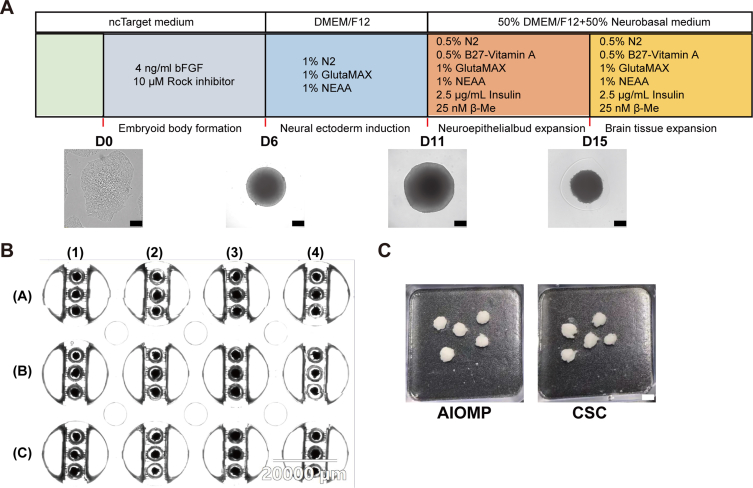
Differentiation procedure and bright field identification of brain organoids in AIOMP and CSC groups. (A) Schematic diagram of hBOs culture procedure. (B) In situ brightfield scans of D35 brain organoids cultured in AIOMP. Scale bar, 2000 μm. (C) Images of brain organoids generated in AIOMP and CSC immersed in OCT within embedding cassette. Scale bar, 3 mm.

## Data Availability

Data will be made available on request.
