# Supplementary material for: All-in-one generation and multiomic profiling of human whole brain organoid on a millifluidic plate
Source: Mater Today Bio. 2025 Dec 9;36:102653. doi: 10.1016/j.mtbio.2025.102653 (PMC12767812; doi:10.1016/j.mtbio.2025.102653)
Supplement: Multimedia component 1 [file mmc1.docx]

Supplementary Materials

**All-in-one generation and multiomic profiling of human whole brain organoid on a millifluidic plate**

Wen Zhao ^a, b, 1^, Yu Wang ^c, d,1^, Tao Chen ^b^, Min Shen ^e^, Jibo Wang ^b^, Xuemei Huang ^b^, Lili Zhu ^b^, Ting Yu ^f^, Zhentao Zhang ^f^, Yunhuang Yang ^c, d^, Maili Liu ^c, d^, Dong Wang ^e^, Weihua Huang ^g^, Rui Hu ^c, d^*, Pu Chen ^a, b^**

Table S1.

Materials Used for organoid culture

| Regent or resource | Source | Identifier |
| --- | --- | --- |
| Chemicals, Peptides, and Recombinant Proteins | | |
| ncTarget hPSC Medium | Nuwacell | Cat. #RP01020 |
| bFGF | Peprotech | Cat. #100-18B |
| ROCK inhibitor Y27632 | Stem Cell Technologies | Cat. #72304 |
| DMEM/F12 | Gibco | Cat. #11330032 |
| N2 supplement | Invitrogen | Cat. #17502048 |
| Non-Essential Amino Acids | Gibco | Cat. #11140050 |
| GlutaMAX | Gibco | Cat. #35050061 |
| Heparin | Sigma | Cat. #H3149 |
| Neurobasal medium | Gibco | Cat. #21103049 |
| N2 supplement | Gibco | Cat. #17502048 |
| B27 supplement without vitamin A | Gibco | Cat. #12587010 |
| Human insulin | Sigma | Cat. #I9278-5ML |
| beta-mercaptoethanol | Merck | Cat. #8057400005 |
| B27 supplement with vitamin A | Gibco | Cat. #17504044 |

Table S2.

Primer sequences used for RT-qPCR.

| **Genes** | **Forward Primers** | **Reverse Primers** |
| --- | --- | --- |
| *Tuj1* | TGATGCGGTCGGGATACTC | TGGGCCAAGGGTCACTACAC |
| *MAP2* | CAGGAGACAGAGATGAGAATTCC | CAGGAGTGATGGCAGTAGAC |
| *GFAP* | ACTGGCAGAGCTTGTTAGTG | AGTGACAGGAAGAGGTGAGA |
| *SOX2* | AAAATCCCATCACCCACAGCAA | AAAATAGTCCCCCAAAAAGAAGTCC |
| *Nestin* | AGCGTTGGAACAGAGGTTGGAG | GGCTGAGGGACATCTTGAGGTG |
| *ISL1* | GCTTTGTTAGGGATGGGAAA | ACTCGATGTGATACACCTTGGA |
| *PAX2* | CTGGGCAGCAACGTGTCA | GAGTGGTGCTCGCCATGTC |
| *PAX6* | AGTTCTTCGCAACCTGGCTA | ATTCTCTCCCCCTCCTTCCT |
| *FOXG1* | AGGAGGGCGAGAAGAAGAAC | TGAACTCGTAGATGCCGTTG |
| *TNF-α* | GTGAGGAGGACGAACATC | GAGCCAGAAGAGGTTGAG |
| *IL-6* | TGAGAGTAGTGAGGAACAAG | CGCAGAATGAGATGAGTTG |
| *IL-10* | TGGAGCAGGTGAAGAATG | TCTATGTAGTTGATGAAGATGTC |
| *IL-4* | CCTCTGTTCTTCCTGCTA | AGATGTCTGTTACGGTCAA |
| *GAPDH* | GGACCTGACCTGCCGTCTAG | GTAGCCCAGGATGCCCTTGA |

Table S3.

Antibodies used for Histology and immunofluorescence.

| Antibody | Source | Identifier |
| --- | --- | --- |
| Anti-Ki67, Mouse, 1:200 | Cell Signaling | Cat. #9449 |
| Anti-MAP2, Rabbit, 1:200 | Cell Signaling | Cat. #4542 |
| Anti-Tuj1, Mouse, 1:400 | Biolegend | Cat. #801201 |
| Anti-Tuj1, Rabbit, 1:200 | Cell Signaling | Cat. #5568 |
| Anti-TBR1, Rabbit, 1: 200 | Cell Signaling | Cat. #49661 |
| Anti-PAX6, Rabbit, 1:200 | Cell Signaling | Cat. #60433 |
| Anti-ISL1, Mouse, 1:200 | Invitrogen | Cat. #MA5-15516 |
| Anti-SOX2, Rabbit, 1:200 | Boster | Cat. # M00105-1 |
| Anti-Nestin, Mouse, 1:200 | Cell Signaling | Cat. #33475 |
| Anti-GFAP, mouse, 1:200 | Invitrogen | Cat. #MA5-12023 |
| Anti-Mouse IgG (H+L) Secondary Antibody Alexa Fluo 568, Donkey, 1:500 | Invitrogen | Cat. #A10037 |
| Anti-Mouse IgG (H+L) Secondary Antibody Alexa Fluo 488, Donkey, 1:500 | Invitrogen | Cat #A21202 |
| Anti- Rabbit IgG (H+L) Secondary Antibody Alexa Fluo 568, Donkey, 1:500 | Invitrogen | Cat. #A10042 |
| Anti- Rabbit IgG (H+L) Secondary Antibody Alexa Fluo 488, Donkey, 1:500 | Invitrogen | Cat. #A21206 |
